# Successful treatment of intractable gastrointestinal tract graft-vs-host disease with oral beclomethasone dipropionate in pediatric and young adult patients: Case reports

**DOI:** 10.1097/MD.0000000000029054

**Published:** 2022-03-18

**Authors:** Koshi Akahane, Atsushi Watanabe, Shinpei Somazu, Daisuke Harama, Tamao Shinohara, Shin Kasai, Hiroko Oshiro, Kumiko Goi, Norio Hasuda, Chihiro Ozawa, Kanji Sugita, Takeshi Inukai

**Affiliations:** ^a^ *Department of Pediatrics, Faculty of Medicine, University of Yamanashi, Chuo, Yamanashi, Japan,* ^b^ *Department of Pediatric Surgery, Faculty of Medicine, University of Yamanashi, Chuo, Yamanashi, Japan,* ^c^ *Department of Pharmacy, University of Yamanashi Hospital, Chuo, Yamanashi, Japan.*

**Keywords:** allogeneic hematopoietic stem cell transplantation, beclomethasone dipropionate, gastrointestinal tract graft-vs-host disease, pediatric and young adult patients

## Abstract

**Rationale:**

The gastrointestinal (GI) tract is a common target organ of graft-vs-host disease (GVHD) in hematopoietic stem cell transplantation (HSCT) patients, and GI tract GVHD is often resistant to standard treatments such as corticosteroids. Moreover, longterm use of systemic corticosteroids sometimes induces adverse events such as infection. Beclomethasone dipropionate (BDP) is a potent, topically active corticosteroid, which is metabolized to an active derivative in the intestinal mucosa. Oral BDP therapy is reportedly effective against GI tract GVHD in adult HSCT patients, but its efficacy and safety in pediatric patients remain undefined. Here, we report three pediatric and young adult cases who were treated with oral BDP.

**Patient concerns:**

Three (6-, 7-, and 18-year-old) patients developed stage 2 to 4 lower GI tract GVHD, which was resistant to standard immunosuppressive therapies.

**Diagnosis:**

Lower GI tract GVHD in these patients was histopathologically proven by endoscopic biopsy.

**Interventions:**

Oral administration of enteric-coated capsules of BDP (3-8 mg/day) was started for the treatment of lower GI tract GVHD.

**Outcomes:**

With the introduction of oral BDP therapy, their GI tract symptoms promptly resolved (abdominal pain, within 3-7 days; diarrhea, within 2-3 weeks). Subsequently, systemic immunosuppressive agents such as corticosteroids and mycophenolate mofetil were successfully tapered off. During oral BDP therapy, although cytomegalovirus antigenemia and *Acinetobacter Iwoffii* sepsis developed in 2 cases, both were curable with conventional treatments. In a young adult case, concomitant BK virus-associated hemorrhagic cystitis resolved after oral BDP was introduced and systemic immunosuppressive agents were reduced. Transient growth restriction was observed in a pediatric case who was treated with oral BDP for approximately 300days.

**Lessons:**

Our experiences suggest that oral BDP therapy is an effective approach for GI tract GVHD that is resistant to standard immunosuppressive therapies. Of clinical importance, our case suggests the possibility that oral BDP therapy may improve the immunosuppressive condition in GI tract GVHD patients by contributing to the reduction of systemic immunosuppressive medications as a result of prompt improvement of GI tract GVHD symptoms.

## 1. Introduction

Graft-vs-host disease (GVHD) is a major cause of morbidity and mortality after allogeneic hematopoietic stem cell transplantation (HSCT). Since the gastrointestinal (GI) tract is a common target organ of GVHD, control of the disease is clinically important. There are 2 categories of the GI tract GVHD: upper GI tract GVHD and lower GI tract GVHD. Typical clinical symptoms of the upper GI tract GVHD include anorexia and nausea, whereas those of the lower GI tract GVHD include crampy abdominal pain and diarrhea. GI tract GVHD is histopathologically characterized by lymphocyte infiltration and crypt epithelial cell apoptosis in the absence of other inflammatory or infectious causes. To control GI tract GVHD, systemic administration of corticosteroids, such as prednisone at 1.0 to 2.0 mg/kg/day, is widely used in combination with prophylactic immunosuppressive agents such as calcineurin inhibitors and methotrexate (MTX).^[1]^ However, approximately two-thirds of patients with acute GI tract GVHD are reportedly resistant to these standard treatments.^[2]^ In addition, since long-term administration of systemic corticosteroids is usually required to control GI tract GVHD, most cases with GI tract GVHD, particularly children and adolescents, suffer from adverse events including infection, adrenal insufficiency, glucose intolerance, bone demineralization, and growth restriction.

Beclomethasone dipropionate (BDP) is a potent, topically active corticosteroid.^[3,4]^ BDP is metabolized to active beclomethasone 17-monopropionate (17-BMP) by esterase enzymes in the intestinal mucosa and bronchial mucosa. Of clinical importance, anti-inflammatory activity of BDP is reportedly 500 to 5000 times higher than that of dexamethasone and hydrocortisone.^[3,4]^ The efficacy of orally administered BDP has been confirmed in the treatment of inflammatory bowel diseases.^[5,6]^ Moreover, systemic adverse effects of orally administered BDP have been considered to be limited due to incomplete mucosal absorption and rapid metabolic inactivation in the liver.^[7]^ Under these circumstances, oral administration of BDP is reportedly effective for mild-to-moderate GI tract GVHD in adult HSCT patients, either as a monotherapy or in combination with systemic corticosteroids.^[8-12]^ However, its efficacy and safety in pediatric HSCT patients remain undefined. Here, we report three pediatric and young adult cases with intractable GI tract GVHD, who were successfully treated with oral administration of BDP.

## 2. Case reports

### 2.1. Case 1

A 7-year-old boy with metastatic (stage M) neuroblastoma (undifferentiated type, *MYCN*-amplified) underwent unrelated cord blood transplantation from a 3/6 human leukocyte antigen (HLA)-matched (mismatches in HLA-A, HLA-B, and HLA-DRB1 loci) and killer-cell immunoglobulin-like receptor ligand (HLA-C)-mismatched male donor after 5 courses of chemotherapy, surgical resection of primary tumor, irradiation to residual bone metastasis, and subsequent autologous peripheral blood stem cell transplantation following high-dose chemotherapy [melphalan (MEL), etoposide, and carboplatin]. After a preparative regimen consisting of fludarabine (FLU) (25 mg/m^2^/day for *5* days), MEL (70mg/m^2^/day for 2 days), and total body irradiation (TBI) 2Gy, unrelated cord blood cells (12.7 × 10^7^/kg of mononuclear cells) were transfused with GVHD prophylaxis of tacrolimus (TAC) and short-course MTX. Neutrophil engraftment (neutrophil counts over 500/μL) was observed on day 24, and full donor chimerism in peripheral blood cells was confirmed on day 36 by microsatellite analysis. The patient developed crampy abdominal pain and watery diarrhea on day 20 and bloody diarrhea on day 35 (Fig. 1). Endoscopic examination and biopsy of the rectum on day 37 revealed a typical finding of GVHD, and a clinical diagnosis of grade III acute GVHD (skin: stage 1; gut: stage 4) was made. After methylprednisolone (mPSL) pulse therapy (25mg/kg/day for 3 days) followed by moderate doses of mPSL (2 mg/kg/day or less) in combination with mycophenolate mofetil (MMF) and weekly administration of MTX, his bloody diarrhea gradually resolved, but his crampy abdominal pain and watery diarrhea still persisted. He also developed BK virus-associated hemorrhagic cystitis and had moderate renal dysfunction probably due to TAC. Considering his persistent GI tract symptoms due to the lower GI tract GVHD, we started oral administration of entericcoated capsules of BDP (3 mg/day, 3 times daily) on day 94 under approval by the ethics committee of the University of Yamanashi with written consent from the parents. His abdominal pain resolved within 3 days after the start of oral BDP, and his watery diarrhea improved within 3 weeks. Then, systemic administration of MTX, mPSL, and MMF was gradually tapered off. Although the patient had cytomegalovirus (CMV) antigenemia from day 133 to day 210, it was successfully treated with ganciclovir and foscarnet, and CMV enterocolitis did not develop. Oral BDP began to be tapered from day 200 and it was discontinued on day 323. The patient had no sign of recurrence of GI tract GVHD until 48 months after unrelated cord blood transplantation.

**Figure:**
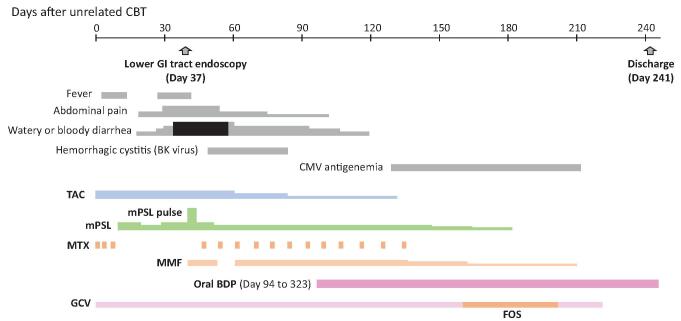
**Figure 1.** Clinical course of case 1.

### 2.2. Case 2

An 18-year-old boy with idiopathic severe aplastic anemia underwent unrelated bone marrow transplantation (BMT) from a 4/6 HLA-matched (mismatches in HLA-A and HLA-DRB1 loci) male donor after 9 years of treatment with cyclosporin A. After a preparative regimen consisting of FLU (25 mg/m^2^/day for 5 days), MEL (70mg/m^2^/day for 2 days), rabbit anti-thymocyte globulin (ATG) (1.25 mg/kg/day for 4 days), and TBI 2Gy, unrelated bone marrow cells (2.0 × 10^8^/kg of mononuclear cells) were transfused with GVHD prophylaxis of TAC from day -1. However, TAC was discontinued on day 2 as encephalopathy developed. Ultimately, GVHD prophylaxis was implemented with MMF, mPSL, and short-course MTX and his encephalopathy subsided. Neutrophil engraftment was observed on day 24, and full donor chimerism in peripheral blood cells was confirmed on day 36 by microsatellite analysis. The patient developed crampy abdominal pain and watery diarrhea on day 32 and bloody diarrhea on day 34 (Fig. 2). Endoscopic examination and biopsy of the descending colon, sigmoid colon, and rectum on day 37 revealed a typical finding of GVHD, and a clinical diagnosis of grade III acute GVHD (gut: stage 3) was made. He simultaneously developed BK virus-associated hemorrhagic cystitis. Escalating doses of mPSL to 2mg/kg/day failed to improve his GI tract symptoms and exacerbated his hemorrhagic cystitis. Under these circumstances, we started oral administration of enteric-coated capsules of BDP (8 mg/day, 4 times daily) on day 44 under approval by the ethics committee of the University of Yamanashi with written consent from the parents. His abdominal pain resolved within 7 days after the start of oral BDP, and his diarrhea gradually improved within 2 weeks. Systemic administration of mPSL and MMF was tapered, and his BK virus-associated hemorrhagic cystitis resolved around day 60. On day 76, the patient developed *Acinetobacter Iwoffii* sepsis, and it was successfully treated with antibiotics. Oral BDP was discontinued on day 85. The patient had no sign of recurrence of GI tract GVHD until 64 months after unrelated BMT.

**Figure:**
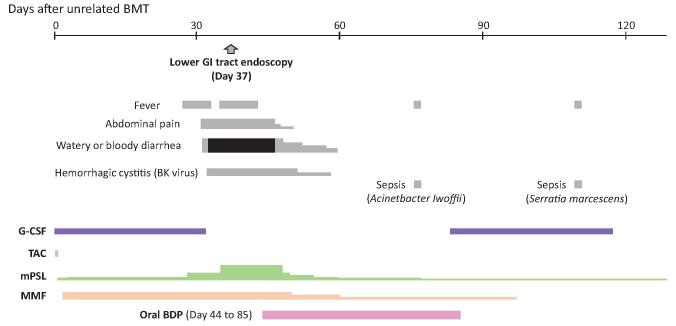
**Figure 2.** Clinical course of case 2.

### 2.3. Case 3

A 6-year-old girl with idiopathic severe aplastic anemia underwent unrelated BMT from a 4/6 HLA-matched (mismatches in HLA-A and HLA-DRB1 loci) male donor after 2 courses of immunosuppressive therapy consisting of rabbit ATG and cyclosporin A in combination with eltrombopag. After a preparative regimen consisting of FLU (25 mg/m^2^/day for 4 days), MEL (70 mg/m^2^/day for 2 days), rabbit ATG (2.5 mg/kg/day for 2 days), and TBI 3Gy, unrelated bone marrow cells (1.6 × 10^8^/kg of mononuclear cells) were transfused with GVHD prophylaxis of TAC and short-course MTX. Neutrophil engraftment was observed on day 15, and the last platelet transfusion was provided on day 22. Full donor chimerism in peripheral blood cells was confirmed on day 36 by FISH analysis of sex chromosomes. The patient developed grade II acute GVHD (skin: stage 1; gut: stage 1; liver: stage 1) on day 40, which was successfully treated with mPSL (1 mg/kg/day). After dose reduction of prednisolone (PSL) to 0.15 mg/kg/day, the patient developed crampy abdominal pain and watery diarrhea on day 101 (Fig. 3A). An escalating dose of PSL (0.6 mg/kg/day) temporarily improved her GI tract symptoms, but her lower GI tract symptoms deteriorated again around day 130. Endoscopic examination and biopsy of the sigmoid colon and rectum on day 140 revealed a typical finding of GVHD, and a clinical diagnosis of stage 2 GI tract GVHD was made. Considering her lower GI tract GVHD symptoms, which were resistant to systemic corticosteroid, we started oral administration of enteric-coated capsules of BDP (3 mg/day, 3 times daily) on day 148 under approval by the ethics committee of the University of Yamanashi with written consent from the parents. Her abdominal pain resolved within 5 days after the start of oral BDP, and her diarrhea improved within 2 weeks. As her lower GI tract symptoms resolved, systemic administration of PSL was tapered off on day 272. Oral BDP began to be tapered from day 376 and was discontinued on day 453. Although the patient transiently developed growth restriction in height during her oral BDP therapy, catch-up growth was confirmed after the cessation of oral BDP therapy (Fig. 3B). The patient had no sign of recurrence of GI tract GVHD until 37 months after unrelated BMT.

**Figure:**
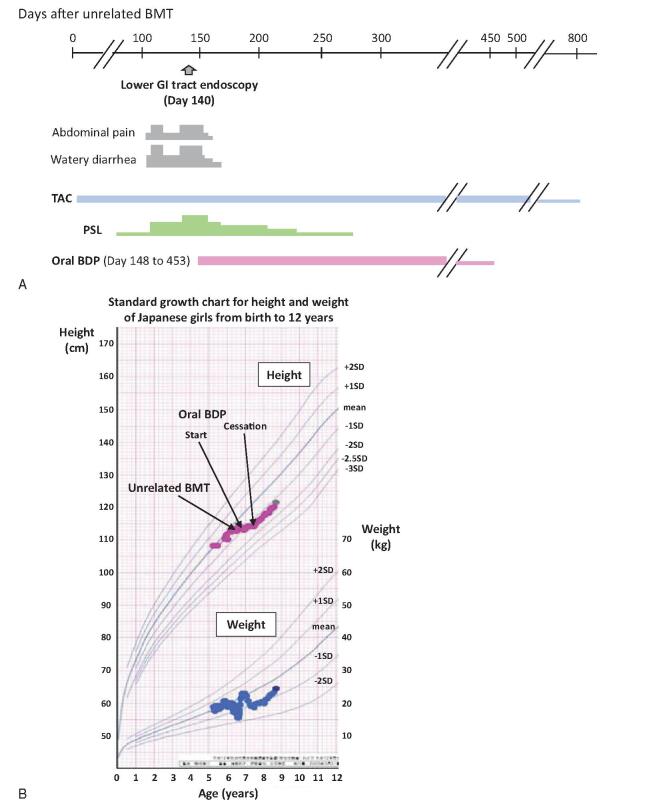
**Figure 3.** (A) Clinical course of case 3. (B) Changes in height (red line) and weight (blue line) during the treatment period in case 3.

## 3. Discussion

Here, we report three consecutive pediatric and young adult cases of GI tract GVHD who were successfully treated with oral administration of BDP (Table 1). All 3 patients received HSCT from two or more HLA loci-mismatched unrelated donors. These patients showed typical lower GI tract symptoms such as crampy abdominal pain and watery or bloody diarrhea without developing typical upper GI tract symptoms. Lower GI tract GVHD in these patients was histopathologically proven by endoscopic biopsy. We treated the 3 patients with enteric-coated capsules of BDP as topical treatment for lower GI tract GVHD. Pharmacologically, enteric-coated capsules are supposed to be dissolved in the small intestine and deliver active BDP into the small intestine and the colon, which are target organs of lower GI tract GVHD.^[8,9,13]^ Although lower GI tract GVHD in these 3 patients was relatively severe (case 1, stage 4; case 2, stage 3; case 3, stage 2) and resistant to conventional immunosuppressive therapies, oral BDP therapy was highly effective. In a previous report of oral BDP monotherapy for adult patients with stage 1 or 2 acute GI tract GVHD, therapeutic responses were confirmed at a median of 6 days (range, 3-14 days) after administration of oral BDP.^[11]^ Consistently, in our 3 pediatric and young adult cases with stage 2 to 4 GI tract GVHD, GI tract GVHD symptoms resolved immediately after the introduction of oral BDP therapy (abdominal pain, 3-7days; diarrhea, 2-3weeks).

**Table 1:** Characteristics and clinical course of presented patients.

	**Case 1**	**Case 2**	**Case 3**
Age (years)/Sex	7/M	18/M	6/F
Disease	Neuroblastoma	Aplastic anemia	Aplastic anemia
Stem cell source	Unrelated cord blood	Unrelated bone marrow	Unrelated bone marrow
HLA match (allele)	3/6	4/6	4/6
Conditioning regimen	FLU + MEL + TBI 2 Gy	FLU + MEL + rabbit ATG (total 5.0 mg/kg) + TBI 2Gy	FLU + MEL + rabbit ATG (total 5.0mg/kg) + TBI 3Gy
Total infused nucleated cells (cells/kg body weight)	12.7 × 10^7^	2.0 × 10^8^	1.6 × 10^8^
GVHD prophylaxis	TAC + MTX	MMF + MTX + mPSL	TAC + MTX
Onset of GI tract GVHD	Day 20	Day 32	Day 101
Severity of GI tract GVHD	Stage 4	Stage 3	Stage 2
Severity of co-existing GVHD	Skin: stage 1	None	None
Treatment for GVHD before the start of oral BDP	TAC + MTX + mPSL + MMF	MMF + mPSL	TAC + PSL
Start date of oral BDP	Day 94	Day 44	Day 148
Therapeutic response	Effective	Effective	Effective
Period from the start of oral BDP to improvement of abdominal pain (days)	3	7	5
Period from the start of oral BDP to improvement of diarrhea (weeks)	3	2	2
Duraion of oral BDP administration (days)	229	41	305
CMV disease during treatment with oral BDP	CMV antigenemia	No	No
Other infectious complications during treatment with oral BDP	No	Sepsis (*Acinetobacter Iwoffii*)	No
Potential adverse event associated with oral BDP (other than infectious complications)	No	No	Growth restriction
Recurrence of GI tract GVHD after the cessation of oral BDP	No	No	No
Survival	Alive	Alive	Alive

In a previous pharmacokinetic study of oral BDP in 12 healthy adult subjects, 17-BMP, an active metabolite of BDP, was detected at low concentrations in the plasma following oral administration of 4mg of BDP.^[14]^ Furthermore, in a phase I study of oral BDP for adult patients with mild-to-moderate GI tract GVHD, transient suppression of hypothalamic-pituitary-adrenal axis was observed in 11 (55%) out of 20 patients whose baseline adrenal function was normal.^[8]^ These observations suggest that the active metabolite of oral BDP (17-BMP) may show some systemic effects in GI tract GVHD patients. In this regard, transient growth restriction was observed in one of our cases (case 3), who was administrated with oral BDP for approximately 300 days. On the other hand, in a phase I study of oral BDP monotherapy for treatment of acute GI tract GVHD, 13 (50%) of 26 patients developed 1 or more infectious episodes during the first 100 days after HSCT.^[11]^ Another study revealed a high incidence of CMV antigenemia (9/15, 60%) and CMV enteritis (3/15, 20%) in GI tract GVHD patients treated with oral BDP.^[15]^ Meanwhile, in the other studies, the incidence of fungal or bacterial infection after HSCT was not increased in GI tract GVHD patients treated with oral BDP.^[8,9]^ In our cases, case 1 developed CMV antigenemia and case 2 developed *Acinetobacter Iwoffii* sepsis during oral BDP therapy. However, these infectious complications were curable with conventional treatment in both cases. Of clinical importance, case 2, who suffered from BK virus-associated hemorrhagic cystitis before administration of oral BDP, was successfully treated after oral BDP was introduced and other systemic immunosuppressive agents were reduced. Thus, although oral BDP itself has some immunosuppressive effects, oral BDP therapy may improve the immunosuppressive condition in GI tract GVHD patients by contributing to the reduction of systemic immunosuppressive medications as a result of prompt improvement of GI tract GVHD symptoms.

In conclusion, our experiences with 3 pediatric and young adult cases suggest that oral BDP therapy is an effective approach for GI tract GVHD that is resistant to standard immunosuppressive therapies. Of clinical importance, our cases suggest the possibility that oral BDP may improve the immunosuppressive condition by contributing to the reduction of systemic immunosuppressive medications. However, the prolonged administration of oral BDP could have some growth impairment effects in pediatric cases. Further clinical studies are urgently required to investigate the efficacy and safety of oral BDP for refractory GI tract GVHD in pediatric and young adult cases.

## Acknowledgments

The authors would like to thank Aya Ichinose and Masahiko Suzuki for the preparation of the enteric-coated capsules of BDP.

## Author contributions

**Conceptualization:** Kumiko Goi, Kanji Sugita.

**Investigation:** Koshi Akahane, Atsushi Watanabe, Shinpei Somazu, Daisuke Harama, Tamao Shinohara, Shin Kasai, Hiroko Oshiro, Kumiko Goi, and Norio Hasuda.

**Methodology:** Chihiro Ozawa.

**Supervision:** Kumiko Goi, Kanji Sugita, Takeshi Inukai.

**Writing** - **original draft**: Koshi Akahane.

**Writing** - **review & editing:** Takeshi Inukai.
